# Alarming update on incidence of Crimean-Congo hemorrhagic fever in Iraq in 2023

**DOI:** 10.1016/j.ijregi.2023.11.018

**Published:** 2023-11-26

**Authors:** Zeenah Atwan, Riyad Alhilfi, Alaa K Mousa, Salman Rawaf, Juan D.L. Torre, Ali R Hashim, Inas K Sharquie, Hanan Khaleel, Celine Tabche

**Affiliations:** 1 Central Laboratory, College of Medicine, University of Basrah, Basrah, Iraq & WHO Collaborating Centre for Public Health Education and Training, Imperial College London, UK; 2 Directorate of Public Health, Ministry of Health, Baghdad, Iraq; 3 Department of Internal Medicine, College of Medicine, University of Basrah, Basrah, Iraq; 4 Public Health Director, WHO Collaboration Center, Imperial College, London, UK; 5 Professor of Immunology & Microbiology, Scripps Research Institute, San Diego, CA, USA; 6 Department of Immunology & Microbiology, College of Medicine, University of Baghdad, Baghdad, Iraq; 7 Surveillance section, Communicable Diseases Control Center, Directorate of Public Health, Ministry of Health, Baghdad, Iraq; 8 WHO Collaborating Centre for Public Health Education and Training, Imperial College London, UK

**Keywords:** Crimean-Congo hemorrhagic fever (CCHF), Virus outbreak, Iraq, Ticks, Infectious disease, Infectious diseases

## Abstract

**Objectives:**

In 2021, large outbreak of Crimean-Congo hemorrhagic fever (CCHF) was reported in Iraq and cases have increased without any significant control measures. To raise awareness about the increasing cases in different regions of Iraq, hence remind the necessity to tackle contributing factors and potential outbreak interventions.

**Methods:**

The study included 511 polymerase chain reaction-confirmed CCHF infection cases out of 1827 suspected cases from 18 Provinces from January to August 2023. Approval from the Ministry of Health for data analyzed.

**Results:**

Out of 1827 suspected cases, 511 were confirmed positive by polymerase chain reaction. The total case fatality rate (CFR) was 12.7 with varying severity levels among provinces. Erbil had the highest CFR, 38.5, while Sulaimaniya and Anbar report no deaths. Independent *t*-test showed a significant difference in CFR between provinces west and south of Baghdad compared to north (*P* <0.05). Trend showed significant surges after Iftar and Adha holidays.

**Conclusion:**

Differences in CFR among provinces around the religious ceremonies, highlight the need for one public health intervention strategy. Increased temperatures affected vector behavior. Uncontrolled animal movement with neighboring countries is an important factor. Virus or host determinants can shape the clinical case outcomes, which need clinical and extensive laboratory studies to unravel the reasons leading to death.

## Highlights

• Crimean-Congo hemorrhagic fever cases surged from 33 in 2021 to 511 by August 2023, signaling a rapid rise.
• Variances in fatality among provinces, highlight the need for tailored interventions.
• Cases spiked during holidays, suggesting cultural practices contribute to the spread.
• Outbreak-linked factors: vector movement, climate change, and free animal trade.
• There is a need for targeted public health actions and international collaboration.

## Introduction

Congo hemorrhagic virus was first isolated and described from patient blood samples in Congo in 1967. An similar antigenic virus was identified in Crimea in 1969, called Crimean-Congo hemorrhagic fever virus (CCHFV) [1]. CCHFV belongs to the Orthonairovirus genus in the Nairoviridae family, which are enveloped viruses with spherical virions and transmitted by ticks [2], [3], [4]. The virus has a tri-segmented negative-sense RNA strand. The large (L) segment encodes for the viral RNA-dependent RNA polymerase. The medium (M) segment encodes for two virus surface glycoproteins, Gn and Gc. The small (S) segment encodes for the nucleocapsid protein (N) [5,6].

CCHFV is transmitted by a *Hyalomma sp.* and since its discovery has spread to more than 30 countries. CCHFV is an endemic in Africa, the Middle East, Eastern Europe, and Central Asia. Recently, CCHFV was also detected in Spain and Southwest Europe and the sequencing analysis showed they were closely genetically related to CCHFV isolates from West Coast of Africa, Mauritania, and Senegal [7], [8], [9], [10]. CCHF outbreaks are mainly driven by *H. marginata* and *anatolicum* due to their high number throughout the year or their higher affinity and aggressiveness to human blood [11].

The ground-feeding birds can serve as carriers of CCHFV-infected ticks, and although they do not transmit the virus to humans, they can facilitate the movement of CCHFV between continents [11]. The annual migration of birds from Africa to the southwest of Europe transferred *H. marginatim* larvae that live on those birds [9,11]. *Hyalomma sp.* are migrating to new areas mainly due to bird migration and climate changes, with some species reaching Germany [12]. Globally, acute CCHF prevalence is 22% with a case fatality rate (CFR) of 11.7% [13].

Iraq has had a history of sporadic cases of CCHF since 1979, and CCHFV was first isolated in 1981 from a limited outbreak of eight patients and two health workers [14]. Sporadic CCHF outbreaks occurred from the 1980s till 2010, with zero to six confirmed cases reported between 1998 and 2009. The suspected number of CCHF cases increased to 28, with 11 laboratory-confirmed cases in 2010. Most small outbreaks were limited to the same province [15]. CCHFV infections have recently increased from 33 cases in 2021 to a 6-fold increase in the first half of 2022 [16,17], underscoring the urgent need for actions to control the disease. This study aims to highlight the increased occurrence, fatality rates, and probable reasons with careful follow-up on the current genotype circulating in Iraq.

## Methods

The study describes reverse transcription-polymerase chain reaction (RT-PCR) confirmed CCHF cases in Iraq from January 01, 2023 till August 31, 2023. Following completion of the RT-PCR test, results are documented, reported accordingly and a copy of each is disseminated to the concerned province. The data was collected from suspected CCHF cases submitted to the Communicable Diseases Control Centre Surveillance section from all 18 provinces of Iraq. Patients with non-specific clinical symptoms including fever, myalgia, headache, vomiting, abdominal pain and a tendency to bleed accompanied by a history of animal contact. A panel of laboratory indicators of low platelets, leukopenia, leukocytosis, prolonged prothrombin time, elevated liver enzyme and impaired renal are critically based as preliminary evidence to be highly suspected and recommended to be tested by PCR. Blood samples are collected by trained professionals from Public Health Department who are the only health personnel allowed to contact the patients. Arabic patient consent forms were signed under the ethical approval of the Public Health Department. Samples are packed and sent immediately to Baghdad, sera are used to do a primary anti-CCHF/IgM and the confirming CCHF virus-specific RT-PCR. Data was collected under a formal ethical agreement and supervision of the Ministry of Health, Iraq. All the data was analyzed and presented using Excel.

## Results

Out of 1827 reported suspected CCHF cases, 511 were confirmed by RT-PCR. The total CFR was 12.7 (65 deaths among 511 confirmed cases). The confirmed cases were higher in provinces west and south of Baghdad, reaching 70%.

Analysis of the CFR among all Iraqi provinces showed differences in the severity of CCHF among the different regions. Baghdad and cities to the north of it displayed the highest CFR among confirmed cases: Erbil 38.5%, Dahuk 25%, Kirkuk 22.2%, Medical City 18.8%, Salah aldin 16.7%, Diyala 16.7%, Baghdad Karkh 15%, Baghdad Resafa 14.3%, and Nineveh 14.3%. Provinces west and south of Baghdad showed lower CFR, Maysan 3.7%, Wasit 6.5%, Babylon 8%, Thiqar 10%, Muthann 11.4%, and Basra 12%. Anbar and Sulaimaniya reported no deaths among confirmed cases. An independent *t*-test was conducted to detect whether there is a significant difference in CFR between provinces to the west and south of Baghdad and those to the north. A significant difference in CFR between the two groups at *P* = 0.012283 was found (Tables 1 and 2).

**Table 1:** Case fatality rate of Crimean-Congo hemorrhagic fever infection in each Iraqi Province.

Province	Confirmed	Deaths confirmed	Case fatality rate confirmed
Dahuk	8	2	25
Baghdad-Karkh	20	3	15
Wasit	31	2	6.5
Baghdad-Resafa	21	3	14.3
Muthanna	35	4	11.4
Maysan	27	1	3.7
Najaf	22	3	13.6
Babylon	25	2	8
Anbar	2	0	0
Diyala	12	2	16.7
Kirkuk	9	2	22.2
Salah aldin	6	1	16.7
Kerbala	7	1	14.3
Medical city	48	9	18.8
Erbil	13	5	38.5
Sulaymaniya	3	0	0
Basra	83	10	12
Diwaniya	12	2	16.7
Thiqar	120	12	10
Nineveh	7	1	14.3

**Table 2:** Case fatality rate distribution from the north, west, and south provinces of Baghdad.

North Baghdad Provinces		West and South Baghdad Provinces	
Sulaymania	0	Anbar	0
Baghdad R	14.3	Maysan	3.7
Nineveh	14.3	Wasit	6.5
Baghdad Khark	15	Babylon	8
Diyala	16.7	ThiQar	10
Salah Aldin	16.7	Muthanna	11.4
Medical city	18.8	Basra	12
Kirkuk	22.2	Najaf	13.6
Dahuk	25	Kerbala	14.3
Erbil	38.5	Diwaniya	16.7

### Time trend of CCHF cases

**Figure 1:**
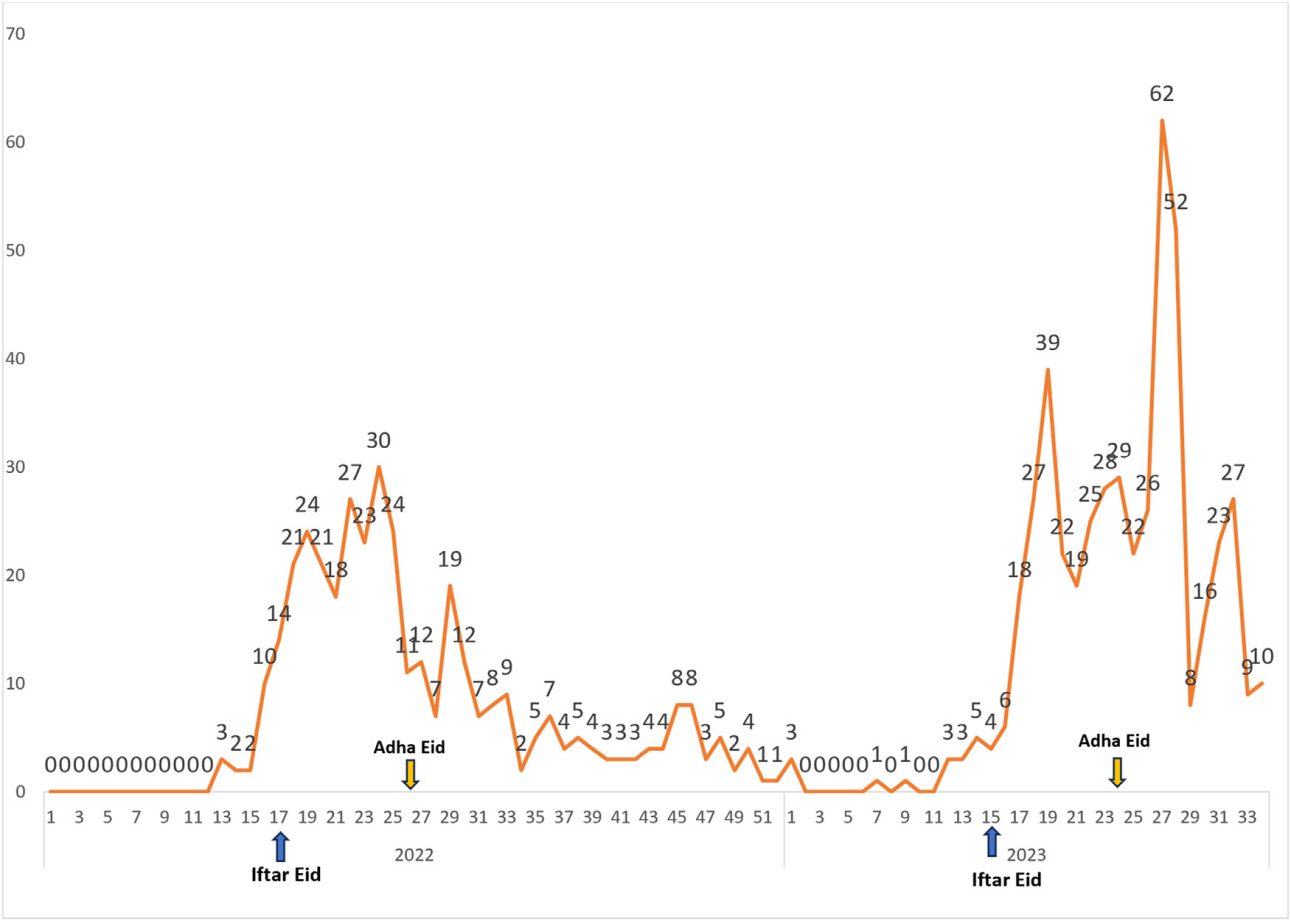
Number of Crimean-Congo hemorrhagic fever confirmed cases in 2023 compared to 2022. Blue arrows refer to Iftar Eid week 17 2022 and week 15 2023. Orange arrows refer to Adha Eid week 26 2022 and week 24 2023.

Analysis of CCHF cases from January to August 2023 revealed a consistent rise in the number of diagnosed cases over time, notably, coinciding with Eid Iftar (15 weeks). There was a substantial surge in CCHF cases, peaking at week 19 before gradually declining. However, compared to the beginning of the year and after the decrease, number of cases remained high. Another prominent increase in diagnosed CCHF cases concurred with Eid Adha reaching the highest at week 27 before it gradually decreased again (Figure 1).

## Discussion

The increased number of RT-PCR-confirmed CCHFV infections in most Iraqi provinces is concerning and emphasizes the need to implement measures to control this upward trend in CCHFV infections to save lives regionally and internationally. Confirmed cases of CCHF increased from 33 in 2021 to 219 in the first half of 2022 to 511 by the end of August 2023. This increase in CCHFV infections in Iraq may be due to the movement of its vector. A recent study showed that *H. anatolicum,* a CCHFV vector, is present in Iraq and was isolated from the skin of CCHFV-infected cattle in the city of Al Dagara, AlDiwanyia province. *H. anatolicum* is genetically related to the Hyalomma species present in Pakistan which has borders with Iran, the Iraqi neighbor [18], [19], [20]. *H. anatolicum* was discovered to be dominant in the South and North of Iran and was found to be at the highest level in deserts, semi-desert areas, and during hot seasons. CCHFV could have also migrated into Iraq from the UAE [18]. CCHFV may have also entered Iraq from other countries in the Middle East, including Turkey. Farmers from different Iraqi provinces, especially at the borders with Turkey and Iran, have reported uncontrolled and illegal entry of sheep and cattle from Turkey, Ukraine, and Iran.

Other factors could have contributed to the increased occurrence of CCHF in Iraq, including increased temperature and climate change. Iraq has witnessed significant temperature and climate fluctuations. *H. marginatum* tends to feed on humans with high aggressiveness and affinity compared to other secondary hosts. This behavior is exacerbated with high (>40°C) temperatures and low humidity [21,22]. Due to the high demand to sacrifice livestock in Adha Eid people tend to obtain needs from private livestock breeders who do slaughtering privately and uncontrolled. Such habit almost occurs all over Iraq and may have contributed to the higher number of CCHF cases.

*H. marginatum* produces 3 to 4.8 generations per year [23,24] and elevated temperature enhances tick reproduction. *H. marginatum* lives and feeds on a wide range of hosts during its lifecycle. It has primary and secondary hosts at immature stages, including wild rabbits, birds, hares, rodents, hedgehogs, and passerine. When they become adults, the species parasitizes camels, cattle, goats, sheep, donkeys, foxes, birds, and humans [25]. Studies examining the genetic variability of CCHF viruses from different outbreaks classified the CCHFV isolates into seven genetically distinct groups, Africa (three groups), Europe (two groups), Asia, including Iraq (two groups) [26,27].

The overall CFR in this study did not exceed 30%, which agrees with other studies. However, some provinces, such as Erbil, showed higher CFR than expected. Analysis of the CFR of provinces around Baghdad revealed a significant decrease in the South, an area where physicians developed a protocol to better identify suspected cases of CCHF based on the patients’ symptoms. Based on previous outbreaks, physicians developed a high level of suspicion to manage the CCHF suspected cases early and effectively. Cases with non-specific symptoms of fever, myalgia, headache, fatigue, vomiting, abdominal pain, sleep disturbance, diarrhea, and tendency to bleed which is in agreement with [28], are considered as potential CCHF cases. In addition, considering occupation, family member infected with CCHF or being in contact with animals are also included. In addition, examining features including bradycardia, bleeding stigmata, organomegaly, and dysfunction of the central nervous system were also taken into account.

Firstly, the physician reflects on a panel of laboratory indicators such as low platelets, leukopenia, leukocytosis, prolonged prothrombin time, elevated liver enzyme and impaired renal are critically based as preliminary evidence to be highly suspected. The suspected case is immediately subjected to infection control measures in terms of house quarantine, explaining the prevention and control measures to relatives and immediate PCR and immunoglobulin (Ig)M tests. Blood samples are taken for PCR and anti-CCHF IgM ELISA. The real-time reverse transcription PCR assay was designed in the most conservative region of the known CCHF groups with a detection level reaching five copies [29]. PCR was of higher significance in the evidence-based decision in detecting virus presence and copy number in serum compared to detection of anti-CCHF-Ig levels. Despite IgM being accurate and reliable in detecting the CCHF, variable sensitivity could result according to the phase of infection [30]. Based on PCR results, the cases were referred to the isolation wards or discharged.

Since ribavirin is effective in reducing the mortality rate in CCHF-confirmed cases, it was the choice of treatment with an efficiency of 80% in treating CCFH-confirmed cases [31]. Mild and moderate cases were treated with ribavirin and discharged after conducting platelets count back to normal within a few days. According to the World Health Organization guidance, ribavirin was given as an initial dose of 30 mg/kg, followed by 25 mg/kg every hour, 6 hours for 4 days, and then 6 days of 7.5 mg/kg every 8 hours [32]. Mild or moderate cases are discharged with recommendation to complete the ribavirin course. A supportive therapy such as steroids was also recommended by the physician for mild, moderate, and severe cases [33].

To manage the critical active hemorrhagic cases, especially those who are in intensive care unit having active bleeding ribavirin injection was mainly prescribed for those who were in coma and could not take the tablets injections.

Time trend analysis showed a notable increase in two time intervals following the Eid Iftar and Eid Adha holidays. This observation correlated with the increased number of controlled and uncontrolled slaughters in all Iraqi provinces. In addition, due to their religious beliefs, people smudge their clothes with the blood of the carcass to make their wishes come true. Other factors, including viral load, route of exposure, and the immediate actions and atypical presentation, could be implicated.

The virus determinants contribution to the severity of infection is unknown. Few studies have revealed genetic variability in CCHF viruses in endemic regions [18,28]. Patients’ genetic factors may be important in shaping the CCHF clinical course such as polymorphism in nuclear factor (NF)-kB, toll-like receptor (TLR)7, and human leukocyte antigen (HLA) alleles that provide insights into immune system function, disease susceptibility, and potential therapeutic targets [34,35].

### Recommendations

To be able to reduce the fatality rate, control infection, and protect public health in Iraq and globally, it is important to take targeted public health measures. First, by ensuring the establishment of sufficient Public Health Labs with full PCR testing facilities across the country. Second, imposing strict measures on unrestrained animal farming and trade. Third, using effective insecticides to control the vectors and leading effective campaigns across Iraq. Since the virus's genetics show a high similarity to its old ancestors, the virus has a very low rate of evolution, and the prospect of developing vaccines will be of great importance. A survey of the tick population could help to identify high-risk communities.

Last, ensuring constant transparency, communication, and collaboration between the public health authorities in the region and globally.

### Limitations

The lack of demographical data interfered with correlating the host determinants with the clinical outcomes. There is a scarcity of studies concerning the virus's genome sequencing and the RNA sequence of the infected patients to be able to suggest the candidate genes involved in the severity of this disease.

## Declarations of Competing Interest

The authors declare that there is no conflict of interest associated with this research study. We have no relationships with individuals or organizations that could influence our objectivity in conducting or reporting this research.
